# Editorial: Plant genotyping: from traditional markers to modern technologies

**DOI:** 10.3389/fpls.2024.1419798

**Published:** 2024-04-30

**Authors:** Yuri Shavrukov, Patricio Hinrichsen, Satoshi Watanabe

**Affiliations:** ^1^ College of Science and Engineering (Biological Sciences), Flinders University, Adelaide, SA, Australia; ^2^ Instituto de Investigaciones Agropecuarias, INIA-La Platina, Santiago, Chile; ^3^ Faculty of Agriculture, Saga University, Saga, Japan

**Keywords:** plant genotyping, traditional markers, modern technology, genome-wide association study (GWAS), simple sequence repeats (SSR), single nucleotide polymorphism (SNP)

Unlike external plant traits, the naked human eye cannot distinguish the genotypes that comprise the underlying genetic material responsible for these phenotypic traits. To make genotypes accessible for research and further understanding and use in plant breeding and related topics, various genotyping methods have become available. Plant genotyping began with quite complex methods based on the direct hybridization of DNA fragments using labelled probes to identify specific genes, which required large quantities of target DNA (as in the case of Restriction fragment length polymorphism, or RFLP). After some years, they evolved into a large series of relatively simpler and cheaper PCR-based methods. These latter reached a peak with very polymorphic and straightforward markers, like microsatellites or SSR (Simple sequence repeats), which were then followed by DNA sequencing and fragment analysis, PCR and qPCR, allele-specific molecular probes and primers, and today’s modern and advanced microchip-DNA technology involving hundreds to thousands of simultaneous reactions.

The current status of our knowledge and progress in plant genotyping was updated in this Research Topic, where we have detailed the available methods and technologies used to target various genes of interest in different plant species. A wide and diverse range of areas were covered and addressed in our Research Topic, from traditional molecular markers to modern microarray technologies. Various scientific approaches and research ideas were incorporated, all aimed at achieving a better understanding of and practical application of plant genotyping. This has led to the resulting 14 published papers that follow.

As mentioned above, SSR markers are a simple, versatile, and straightforward molecular tool for plant genotyping. Yin et al. used SSR markers for practical identification and distinctness testing of a non-heading Chinese cabbage (*Brassica campestris* ssp. *chinensis* Makino). This is a very important test that establishes distinctness, uniformity, and stability (DUS), which are essential factors required for the granting of plant variety rights (PVRs). The authors tested 287 SSR markers for genotyping of 423 non-heading Chinese cabbage varieties, and they used four fluorescent dyes, FAM, HEX, TAMRA and ROX, for the labelling of forward primers. Importantly, two methods were used for scoring, polyacrylamide gel electrophoresis (PAGE) and fluorescence capillary electrophoresis. The resulting 23 core SSR markers were finally selected, enabling perfect genotyping of the majority of the studied non-heading Chinese cabbage varieties. Therefore, a combination of SSR genotyping with simple morphological markers in a field trial provided more accurate and efficient identification of the varieties for the distinctness test. Based on clustering analysis, the authors designated 423 varieties into three Clades and nice coloured photos illustrated their clear distinctness. This is one of the best examples of the simple and elegant application of plant genotyping using SSR markers in crops like non-heading Chinese cabbage.

An oat (*Avena sativa* L.) germplasm collection (132 cultivars and pure lines) with diverse origins was described by Mathias-Ramwell et al. for phenotype and genotype characterization within a Chilean breeding program. Specifically in Chile, a single cultivar (Supernova-INIA) is predominant, covering over 90% of the oat cultivated area. Therefore, this has forced the development of new oat varieties adapted to the changed climate, which is severely affecting the Southern part of Chile. This study combined the evaluation of 28 phenotypic traits and genotyping with 14 SSR markers that were previously reported as informative in oat. The studied oat germplasm collection exhibited a high phenotypic diversity (H’ = 0.68) and grouped into three clusters. This result differed from the SSR-based Structure analysis indicating for the existence of two sub-populations with low genetic distance (0.24), despite moderate (He = 0.58) average genetic diversity. In summary, the combination of both phenotypic data and SSR-based genotyping supported the possibility to obtain genetic gain in the medium to short term in this breeding effort, opening the opportunity for improved oat germplasm materials.

Semalaiyappan et al. focused on pearl millet [*Pennisetum glaucum* (L.) R. Br.; syn. *Cenchrus americanus* (L.) Morrone], a strategic climate-resilient C4 crop and an important staple food in Asia and Africa. The authors retrieved 4K SNPs from 925 whole-genome sequences and carried out genotyping of 373 genetically diverse pearl millet inbred lines. Their genotyping of the SNP panel exhibited a uniform distribution across the entire genome. All studied accessions were effectively designated and differentiated using the SNP panel into two major groups (B and R lines) based on the genetic diversity analysis. The studied 4K SNP panel was reported as very useful for various genomics and molecular breeding applications in pearl millet, including mapping of agronomically important traits and genomic selection.

Genotyping of trees represents a very complicated process and is usually carried out on individuals established and grown over a very long time-frame. However, Wu et al. carried out genetic analysis of 69 parents and 1,793 third-generation offspring (ramets) in the seeds of orchard Chinese fir [*Cunninghamia lanceolata* (Lamb.) Hook]. This was very extensive research involving both morphological and molecular analyses. The authors used traditional SSR markers for plant genotyping to study the mating system and flowering phenology of trees. The SSR genotyping was based on fluorescent labels, FAM or HEX dyes, attached to forward primers. This approach is well known and widely used for plant genotyping, and it was very suitable for this study of Chinese fir trees. The results described genetic co-ancestry among parental genotypes that was detected in the third generation of ramets genotypes. Effective pollination (68.1%) occurred within 50 m, and it was successful if about 30% of male and female flowers overlapped in their flowering. It is important to emphasize that such an accurate and delicate study was achievable through plant genotyping using SSR markers.

Another study of a forest species was presented by Yan et al. The authors reported on the application of SSR markers for genotyping, analysis of genetic diversity and population structure in a collection of 161 Korean pine clones (*Pinus koraiensis* Siebold & Zucc.), originating from seven populations in Northeast China. A set of 77 alleles derived from 11 SSRs would at first seem very small but this was sufficient to accurately distinguish each clone. However, a rather low genetic diversity was exhibited among different populations, but diversity was higher within each studied population, explaining 98% of the total observed variation. This is a very unusual result for genotyping of Korean pine populations. Moreover, only one population, from Lushiuhe, was isolated and differentiated clearly. The set of 11 SSR markers used was proposed as a fingerprinting tool able to identify any specimen of Korean pine, and this final result can be potentially used for the breeding of this species.

Modern high-throughput genotyping microarrays provide thousands of simultaneous reactions, and Ding et al. presented a report on the successful application of 55K SNP microarrays for analysis of time to heading and maturity in a diverse group of 239 bread wheat accessions (*Triticum aestivum* L.). Starting from genome-wide association study (GWAS), the authors carried out three-year experiments in four environments. For genotyping, 16,649 high-quality SNP markers were selected and in the results of GWAS, 238 and 55 SNP markers were found to be strongly associated with time to heading and maturity, respectively. Finally, the authors identified only nine marker-trait associations in different environments with highest scores across the entire group of studied wheat genotypes. This resulted in nine SNPs in the most promising candidate genes controlling traits for time to heading and maturity in bread wheat. Many genes are involved in the control of such important traits as heading and maturity, and the authors discussed functions of these candidate genes in the paper: Zinc transporter and Zinc finger family protein, Glycosyltransferase and S-acyltransferase, F-box protein and Cytochrome P450, Calcium-dependent protein kinase and Photosystem II stability/assembly factor, and Cytokinin phosphoribohydrolase.

A genome-wide association study of sorghum was carried out by Wang et al. focusing on plant colour of sorghum [*Sorghum bicolor* (L.) Moench], which influences various traits such as seed colour as well as disease resistance and phytoalexin production. Using a sorghum mini-core collection, the authors assessed the colour of leaf sheaths and blades across three environments and conducted genome-wide association mapping with 6,094,317 SNP markers. Eight QTLs were identified and linked to plant colour, containing up-to 1-3 candidate genes each. These findings offer insights for the application of plant genotyping for plant colour development and in sorghum molecular breeding.

Two studies of maize (*Zea mays* L.) populations were based on the Iowa Stiff Stalk Synthetic (BSSS) germplasm stock. In the first paper, Ledesma et al. described the molecular characterization of the collection of DH lines derived from the unselected BSSS population (C0) and those after 17 cycles of reciprocal recurrent selection in BSSS (C17). The progenies of a hybrid population between C0 and C17 were genotyped with a set of 24,885 SNP markers distributed among 10 maize chromosomes for evaluation of their genetic variability. The authors also studied the possible loss of genetic diversity during the recurrent selection process from C0 to C17. The reported results confirmed a net loss of variability with the degree of differentiation between C0 and C17 DH groups. The different contribution of the progenitors of DH lines derived from C0, C17 or their hybrid was mostly explained by genome-wide genetic drift. Additionally, complementary to allelic selection occurred during the reciprocal recurrent breeding supported by phenotype analysis data.

The continuation of the previous study with maize was reported by Ledesma et al. The authors applied GWAS for maize plant architecture traits, which were modified during the selection of BSSS populations with a very big impact on grain moisture and yield, root and stock lodging. Using the same approach, the authors compared phenotypes and genotypes of DH lines derived from BSSS recurrent selection. It included C17 DH, reciprocal recurrent selection (R) and from their hybrid. Plant phenotypes and studied agronomic traits as well as identified genes or genomic regions were associated with modifications in the plant architecture. Additionally, plant density and grain yield traits, including flowering time and time from anthesis to silking, showed high heritability and were more common for the BSSS(R)C17 DH lines. Finally, a considerable number of SNPs were identified in the genetic regions with promising candidate genes associated with plant architecture traits using the entire set of DH lines. Therefore, the genetic basis of the studied traits can be elucidated for marker-assisted selection schemes in maize breeding in future.

The effect of nitrogen fertilization levels on three related traits in maize (plant height, grain yield, and time from anthesis to silking) was explored by Sanchez et al., where phenotypic analysis was combined with GWAS for nitrogen use efficiency (NUE). For this purpose, 181 double haploid (DH) maize lines were studied using GWAS with 62,077 SNPs for plant genotyping. For three studied traits, data were collected from conditions of high or low nitrogen, under three environments, for both *per se* and testcross trials. Interestingly, significant genetic variation was observed among the DH lines and their respective testcrosses, using three GWAS models. Additionally, some testcrosses from exotic introgression lines were superior compared to the check hybrid. Finally, some SNPs were associated with agronomic traits under both high and low nitrogen. At the same time, these SNPs belonged to gene models and were related to stress response and nitrogen metabolism. In summary, this SNP-based GWAS analysis revealed the existence of several promising alleles in the maize germplasm panel with genes controlling key agronomic traits.

Genotyping by sequencing (GBS) is another approach for high-throughput plant genotyping. Lu et al. studied resistance of cabbage lines (*Brassica oleracea* L. var. *capitate*) to black rot disease (*Xanthomonas campestris* pv. *campestris*). The authors used GBS for QTL analysis of resistance in the F_2:3_ hybrid population from a cross between resistant (BR155) and susceptible (SC31) parents. The genetic map was established with 7,940 SNP markers, and QTL analysis was carried out for disease resistance in 126 hybrid progenies over three seasons. In the results, the authors reported about seven identified QTLs with only one major QTL, qCaBR1, in chromosome C06. In the genetic interval of the major QTL 96 genes were annotated, but only eight of these genes showed responses to biotic and pathogenic factors. These candidate genes are listed as follows: Chorismate mutase, ß-1,4-N-acetylglucosaminyltransferase, Ethylene receptor, Plastid movement impaired, DNA ligase, Leucine-rich repeat protein kinase, RNA-binding family protein, and Early-responsive to dehydration protein.

Kiwifruit (*Actinidia chinensis* var. *chinensis*) can be attacked by one of the worst all plagues, *Pseudomonas syringae* pv. *actinidiae* (Psa). Therefore, the development of resistant germplasm is always a priority in the breeding of this species. Indeed, seedlings of certain genotypes can be highly susceptible to this disease, reaching up to 100% mortality. Flay et al. approached the search for QTLs associated to resistance to Psa, using a Bulk segregant analysis (BSA) approach. For this purpose, the authors analysed the effect of removing plants with Psa symptoms on the total allele frequency in the produced incomplete-factorial-cross population. The genotype-distinct diploid parents were used in this population consisting of 28 F_1_ families. Only surviving plants from the different families were selected, their DNA was bulked, and QTLs were identified along with their detection accuracy. In addition, each family was assigned to a single bulk grouping according to the genetic contribution of a separate parent to each family. Finally, 11 QTLs were identified based on the deviation of allelic frequencies in the surviving populations in two independent analyses. This information was based on SNPs derived from a 30× bulk sequencing analysis. The authors have used their findings to initiate the development of novel molecular markers applied to the selection of kiwi lines with Psa resistance.

The development of universal markers for plant mitochondrial genomes is challenging because of their variability in size, gene order and sequence conservation. Grosser et al. presented a very interesting report exploring genetic polymorphism in mitochondrial introns to distinguish plant species. This is a very novel and non-traditional approach for differential plant genotyping. The researchers tested PCR primer sets across different angiosperm species and found that amplicon length was much more polymorphic among genera but significantly less within genera. The authors confirmed their results in different plant species. This study emphasized the utility of genetic polymorphism in intron length in the mitochondrial genome across various plant species. The presented results were estimated as providing important and valuable tools for potential applications in evolutionary studies and molecular-genetic research.

Very different was a paper presented by Tran et al., describing a method for single copy transgene identification through qPCR using the example of transgenic rice (*Oryza sativa* L.). The authors established a qPCR protocol for the reference gene *OsSBE4*, encoding starch branching enzyme, and the *nos* terminator used in the transgenic construct. The data reported a near 100% accuracy for the method in distinguishing homozygous single-insert transgenic plants. This assay could be successfully applied to other transgenic rice plants that have the *nos* terminator in their construct. The standard conditions for qPCR can be used with relatively inexpensive dyes, such as SYBR Green. Therefore, the suggested qPCR method could be cost-effective and suitable for lower budget laboratories that are involved in rice transgenic research, or even modified to test transgenics of other species through the selection of primers for a known reference gene that perform comparably to the *nos* primers. The genotyping approach presented in the paper can be targeted not only toward transgene copy number, but also can be used to detect duplication of indigenous genes.

In summary, all 14 papers published in this Research Topic deal with very different aspects of plant biology, ecology, molecular genetics, and genomics, covering different crops and plant species, and quite diverse experimental designs. However, all these papers are united under the single topic of plant genotyping using different types of molecular markers. Some authors used the more traditional SSR markers while others were interested in SNP studies using GWAS and GBS technologies. The last group of researchers were compelled to investigate genetic polymorphism of intron length in mitochondrial genome or methods for the identification of transgene or endogenous gene copy numbers. In this regard, all presented results for plant genotyping are important not only in advancing scientific progress, but for their practical application in crop breeding, supporting biodiversity and biosecurity, and the analysis of plant-derived products for use in food, medicine, or other industries.

## Author contributions

YS: Conceptualization, Writing – original draft. PH: Conceptualization, Writing – original draft. SW: Conceptualization, Writing – original draft.

